# Molar-incisor hypomineralisation prevalence in a cohort of Australian children with type 1 diabetes

**DOI:** 10.1007/s40368-022-00765-z

**Published:** 2022-11-08

**Authors:** C. Lim, E. D. Jensen, B. F. Poirier, S. Sethi, G. Smart, A. S. Peña

**Affiliations:** 1 Adelaide Dental School, University of Adelaide, 4 North Terrace, Adelaide, SA 5000 Australia; 2 Department of Paediatric Dentistry, Women’s and Children’s Hospital, Adelaide, SA Australia; 3 Discipline of Paediatrics and Robinson Research Institute, University of Adelaide, Adelaide, SA Australia; 4 Department of Diabetes and Endocrinology, Women’s and Children’s Hospital, Adelaide, SA Australia

**Keywords:** Type 1 diabetes, T1D, Molar-incisor hypomineralisation, MIH, Children

## Abstract

**Purpose:**

Systemic diseases or drugs administered early in life may cause a disruption in amelogenesis and contribute to the qualitative defect of enamel described as molar–incisor hypomineralisation (MIH). Therefore, an increase in prevalence of MIH in children with type 1 diabetes (T1D) may be expected as this systemic disorder is commonly diagnosed in early childhood. The aim of this study was to determine the prevalence of MIH in a cohort of children with T1D and investigate diagnosis of MIH with T1D factors.

**Methods:**

Cross-sectional study of children with T1D recruited from paediatric diabetes clinics at the Women’s and Children’s Hospital (South Australia). A detailed medical history, comprehensive dental and MIH examination according to the European Academy of Paediatric Dentistry (EAPD) long form classification was collected for each child. All upper and lower first permanent molars and central incisors were scored.

**Results:**

A total number of 73 participants; 35 (47.95%) males were examined including 584 teeth. The mean age of the participants was 13.25 ± 2.58 years, with a mean age of diagnosis 7.75 ± 3.58 years, and a mean HbA1c of 8.5 ± 1.6%. 42 out of 73 children (54.8%) had enamel defects on at least one of the teeth examined. However, 19.2% met the criteria for MIH. Univariate and bivariate analyses were conducted but no significant associations were noted between MIH and risk factors including diabetes control (*p* > 0.1).

**Conclusion:**

There was a high prevalence of enamel defects and MIH amongst children with T1D. More research is required to establish association between T1D and MIH.

## Introduction

Molar-incisor hypomineralisation (MIH) was first described in 2001 as a qualitative defect of enamel presenting as well-demarcated areas affecting the first permanent molars (FPM) and often the permanent incisors (Weerheijm et al. 2001). The defective enamel is characterised by a reduction in mineralisation and inorganic compounds, as well an increase in protein content, resulting in discoloured and brittle enamel (Weerheijm 2004; Farah et al. 2010). The suggestion of a disturbance of ameloblasts during the maturation stage of enamel formation is widely accepted (Weerheijm 2004; Crombie et al. 2009) and hypomineralisation is understood to be a chronological enamel defect, potentially affecting any primary or permanent tooth. The exact cause of the disruption remains unknown, with several suggestions of aetiological factors such as genetics/epigenetics, environmental factors, systemic diseases, or drug exposure during the gestational period or the first three to four years of life (Weerheijm et al. 2001; Crombie et al. 2009; Serna et al. 2016; Silva et al. 2016; Hočevar et al. 2020; Lygidakis et al. 2021).

Type 1 diabetes (T1D) is one of the most common chronic, metabolic diseases that occur in childhood. Diagnosis can occur at any age with a peak between 4 and 7 years of age and during early puberty at 10–14 years of age (Atkinson et al. 2014; Lucier and Weinstock 2022). Type 1 diabetes can cause chronic hyperglycaemia and aggravate oxidative stress, which can affect tissue structure and function of the body (Giacco and Brownlee 2010; Chałas et al. 2016). Protein metabolism in individuals with poorly controlled T1D is also known to be altered with net increased protein breakdown during periods of insulin deprivation (Hebert and Nair 2010). As the potential causative factor(s) remain unknown for MIH and it is known that children with T1D can have chronic metabolic disturbances, exploration of a potential association, particularly if the prevalence is increased in those children diagnosed with T1D at a young age, is of interest (Garot et al. 2021).

Whilst there is evidence that children with chronic health conditions can have increased prevalence of MIH (Mohamed et al. 2021), there are limited studies that explore a potential association between T1D and MIH with those available limited to rodent data. Rats with TID have altered bone and enamel development (Atar et al. 2004; Abbassy et al. 2008, 2010), but literature investigating the association of T1D and MIH in humans is lacking. Therefore, this paper aims to report the prevalence of hypomineralisation and MIH in a population of children with T1D and to investigate the diagnosis of MIH with any associations to T1D parameters.

## Methods

This cross-sectional study recruited consecutively a convenience sample of 73 participants (38 females) with previously diagnosed T1D. Children were recruited from February 2018 to March 2019 through the Women’s and Children’s Hospital (Adelaide, South Australia) as previously detailed in Jensen et al. (2021). Children were eligible for inclusion if aged 8 to 18 years and diagnosed with T1D by detectable islet cells autoantibodies. Children with a diabetes diagnosis other than T1D and those whose English skills hindered the provision of informed consent were excluded from this study. For children under 16 years of age, informed written consent was obtained from parents or guardians; those above 16 years of age provided informed written consent themselves. All children gave assent for the study. This project received ethical approval from the Women’s and Children's Health Network Human Research Ethics Committee (HREC/17/WCHN/165).

### Oral examination protocol

#### Clinical data

Details regarding participant diagnosis and treatment of T1D were obtained from medical records. This included date and age of diagnosis, and HbA1c levels within three months of the dental examination. Participant HbA1c values were measured with the DCA Vantage® Analyzer (Siemens Healthcare Diagnostics, Camberley, UK), which has a high correlation coefficient (*r* = 0.98) with DCCT standardised sample control. Other details such as gestational age, mode of delivery at birth and medical history by systems were also obtained.

#### Molar incisor hypomineralisation

The European Academy of Paediatric Dentistry (EAPD) examination protocol for the diagnosis of MIH (Ghanim et al. 2017) was utilised for this study. Dental examinations were performed by a trained and calibrated practitioner (E.J), using a dental mirror, a ball-ended explorer, and dental chair lighting. A ball-ended explorer was used to examine the teeth for surface irregularities, ensuring any damage on the tooth surfaces was prevented. Participants were advised to brush their teeth prior to the examination. All fully erupted teeth were examined wet to increase the accuracy of results. The long form clinical status of each tooth was recorded. Examination results were correlated with the recommended EAPD MIH diagnosis sequence (Ghanim et al. 2015, 2017). The EAPD classification was measured on the maxillary and mandibular permanent central incisors and FPM.

In accordance with EAPD criteria for MIH diagnosis, each molar and central incisor were examined for the presence, type, and severity of MIH (Ghanim et al. 2015, 2017). The extraction of an FPM due to MIH was identified through past dental records or discussed with the parent/guardian of the child (Weerheijm et al. 2003; Ghanim et al. 2017).

Several considerations for the diagnosis of MIH were used in the examination protocol. Molar–incisor hypomineralisation was diagnosed when at least one molar tooth with MIH was present. Children with affected permanent incisors were not diagnosed with MIH unless there was hypomineralisation present on at least one permanent first molar. If more than one area affected by hypomineralisation was present on a tooth, the higher severity rating was recorded as the main finding for that tooth. A tooth with five-surface full-coverage restoration (in the absence of trauma to an incisor) was considered an atypical restoration and MIH was diagnosed. Post-eruptive enamel breakdown was also recorded when an atypical restoration was missing, and no caries was present. When the tooth meant to be examined for MIH was absent, either congenitally missing or extracted due to causes other than MIH, it was not considered an extraction due to MIH (*n* = 0). Teeth were considered sound when the enamel defect(s) present were diffuse and one millimetre or less in diameter (Ghanim et al. 2015, 2017).

Statistical analyses were performed using SPSS for Windows version 27 (IBM SPSS Inc., Chicago, IL, USA), and included a descriptive evaluation of the results in a bivariate analysis. The association between the presence of MIH and the descriptive variables including the age of diagnosis, type of birth delivery, comorbidities, and HbA1c levels variable was evaluated with the chi-squared association or Fisher test. The level of significance was determined by *p*-value with values less than 0.05 considered significant.

## Results

A total number of 73 participants; 38 females were examined including 584 teeth. Forty-one participants (56.16%) were 8 to 13 years in age and 32 (43.84%) were 14 to 18 years old. Amongst the 41 participants aged less than 14 years, 20 (48.78%) were males; and amongst 32 participants aged more than 14 years, 15 (46.87%) were males (Table 1). The mean age of female and male participants was 13.5 (± 2.5) and 12.92 (± 2.67) years, respectively. The mean age of T1D diagnosis was 7.75 (± 3.75) years for males and 7.75 (± 3.42) years for females and a total of 14 (19.18%) participants had been diagnosed with T1D before 4 years of age. The HbA1c levels ranged from 5.8 to 13.3% (median of 8.0%) amongst participants. Insulin was delivered through pumps for 29 individuals (39.73%). The logistic regression models did not yield any significant results and the individual numbers in each group were quite small due to small sample size of the study. Due to insignificant small numbers the models were not presented and the results were presented in a descriptive manner.

**Table 1:** Distribution of the sample study group according to age and gender

	Sex		Total
	Male	Female	
	Number of participants *n* = 35 (%)	Number of participants *n* = 38 (%)	Number of participants *n* = 73 (%)
Age at examination (years)			
8–13	20 (57.14)	21 (55.26)	41 (56.16)
14–18	15 (42.85)	17 (44.74)	32 (43.84)
Age of T1D diagnosis (years)			
< 4	9 (25.71)	5 (13.16)	14 (19.18)
≥ 4	26 (74.29)	33 (86.84)	59 (80.82)
Delivery^a^			
Vaginal	25 (73.53)	29 (78.38)	54 (76.06)
C-section	9 (26.47)	8 (21.62)	17 (23.94)
HbA1c			
≤ 7%	15 (42.86)	17 (44.74)	32 (43.84)
> 7%	20 (57.14)	21 (55.26)	41 (56.16)
Mode of Insulin Delivery			
Pump	17 (48.57)	15 (39.47)	32 (43.84)
Injection	18 (51.43)	23 (60.53)	41 (56.16)

^a^Two participant’s details unavailable, *n* = 71

*T1D* type 1 diabetes

All 73 participants had upper and lower central incisors and FPM examined (total of 584), a total of 146 upper molar teeth, lower molar teeth, upper incisors and lower incisors, and the findings have been tabulated in Table 2. As per the diagnosis of MIH; in children with at least one affected first permanent molar ± permanent incisors; a prevalence of MIH of 19.18% was found in the study population. The prevalence of children diagnosed with an enamel defect or MIH in the study population have been tabulated in Table 3. The distribution of enamel defects on the teeth examined are detailed in Table 4.

**Table 2:** Distribution of Enamel defects or MIH as per tooth morphology in the study population

Tooth (*N*)	Teeth with enamel defects % (% consistent with MIH by definition)
Upper molars (146)	23.29 (10.27)
Lower molars (146)	25.34 (9.59)
Upper incisors (146)	32.89 (4.79)
Lower incisors (146)	9.59 (2.74)

**Table 3:** Prevalence of children diagnosed and median levels of HbA1c with an enamel defect or MIH in the study population

Condition	Number of children *n* (%)
Total enamel defects (including MIH)	
Present	40 (54.79)
Absent	33 (45.21)
Diagnosis of MIH	
Present	14 (19.18)
Absent	59 (80.82)

*MIH* molar-incisor hypomineralisation

**Table 4:** Distribution of MIH on teeth examined

Classification of enamel defects in long form								
Tooth examined^a^	16	11	21	26	36	31	41	46
1 = Enamel defect, non-MIH								
11 = Diffuse opacities	11	20	19	8	12	5	5	11
21 = Hypoplasia	0	0	1	0	0	0	0	0
13 = Amelogenesis imperfecta	0	0	0	0	0	0	0	0
14 = Hypomineralisation defect on other teeth	0	1	1	0	0	0	0	0
2 = Demarcated opacities								
21 = White/creamy	3	2	1	3	0	3	1	1
22 = Yellow/brown	2	1	2	4	4	0	0	3
3 = Post-eruptive enamel breakdown (PEB)	0	0	0	0	0	0	0	0
4 = Atypical restoration	2	0	0	0	1	0	0	1
5 = Atypical caries	0	0	0	0	0	0	0	0
6 = Missing due to MIH	0	0	0	1	2	0	0	2
7 = Cannot be scored	0	0	0	0	0	0	0	0

^a^Teeth reported as per World Dental Federation notation

A total of 584 teeth were examined and diffuse opacities and hypoplasia were recorded on 32.87% and 9.59% of upper and lower central incisors respectively, as well as 23.29% of upper FPM and 25.34% of lower FPM. Upper central incisors had almost double the MIH when compared to lower central incisors (4.79% and 2.74%).

Glycosylated haemoglobin (HbA1c) levels were used as marker of diabetes control as it measures average glucose levels over the previous 3 months (Carlson et al. 2020). Nine out of 14 of the participants who were diagnosed with MIH had HbA1c levels greater than 7%. However, 64 out of the 73 participants with T1D had HbA1c levels above 7.0%. There was no continuous or categorical association observed (*p* > 0.1) between HbA1c and MIH.

## Discussion

Molar–incisor hypomineralisation was present in 19.2% children and adolescents with T1D, compared to an estimated global prevalence of all healthy children at 13.5% (Lopes et al. 2021). There is no comparative data available regarding the prevalence of MIH amongst Australian children using the MIH classification, but there are population studies of enamel defects in Australian children with a prevalence of 44% in New South Wales (Balmer et al. 2005) and a prevalence of 22% in Western Australia (Arrow 2008) according to the modified developmental defects of enamel (mDDE) index. In comparison to other parts of the world, the prevalence of MIH using the EAPD classification ranges from 6.31% to 20.2% in children under 14 years of age (Martínez Gómez et al. 2012; Zawaideh et al. 2012; Mittal et al. 2013; Ghanim et al. 2014; Amend et al. 2020). The MIH prevalence amongst this cohort falls within the MIH prevalence range of multiple countries (Lygidakis et al. 2021). In this study, 54.79% of participants had some form of enamel defect on one or more FPM or incisors. Despite nine participants having demarcated opacities that were indicative of hypomineralisation on the incisors, only five of the participants also had an affected molar and thus were diagnosed with MIH as per the EAPD classification. Mild MIH was observed in 64% of participants whilst 35% had severe MIH; this is typical of other reported large-cohort studies of healthy children which have reported 40–50% moderate to severe MIH (Martínez Gómez et al. 2012; Ghanim et al. 2014). However, to date there are no studies evaluating MIH in a cohort of children with T1D and healthy children. Gender was not contributory as children with MIH were 50% female. Caesarean-section delivery has been considered a risk factor in the development of enamel defects (Rafatjou et al. 2018). However, 12 out of the 14 participants with MIH were delivered vaginally.

The aetiology of MIH remains unclear, with current hypotheses including disruption to enamel formation during the maturation stage of development by environmental factors, childhood illnesses and during pre, peri, post-natal periods (Crombie et al. 2009; Alaluusua 2012; Bensi et al. 2020; Butera et al. 2021; Garot et al. 2021). The ameloblasts of hypomineralised teeth appear dysfunctional during the early maturation phase (Suga 1989) which occurs from birth to the first 2 years of life for FPM and incisors. Systemic diseases can also have an impact on the cells during the maturation stage (Suga 1989; Jälevik and Norén 2000). The current understanding of the aetiology of T1D is autoimmune destruction of the pancreatic β cells which produce insulin (Todd 2010). Type 1 diabetes is the most prevalent form of diabetes mellitus amongst children. The development of the first autoantibodies amongst individuals who are genetically at risk of diabetes peaked before the age of 2 years (Ziegler et al. 2013; Krischer et al. 2015). Most individuals with a single autoantibody do not proceed into T1D (Ziegler et al. 2013) but the development of the autoantibodies occurs during the first few years of life, which may occur concomitantly with the disturbances of cells during the maturation stage of enamel formation during or before a formal diagnosis of T1D, in which the usual diagnosis of T1D is between 4 and 7 years, or 10 and 14 years (Atkinson et al. 2014; Lucier and Weinstock 2022). The sample population included an average age of T1D diagnosis of 7.75 years of age and 19.18% diagnosed before 4 years of age, limiting the sample for those potentially at higher risk of MIH. Higher HbA1c was anticipated to produce an increased prevalence of MIH but this was not observed.

This study has several strengths. This comprehensive cross-sectional study was conducted using the EAPD MIH classification. This is considered the standardised method of charting MIH (Ghanim et al. 2015) as it incorporates the EAPD criteria (Weerheijm et al. 2003) and the mDDE index (Clarkson and O'mullane 1989). There has historically been a range of different criteria and methods of recording MIH, thus the EAPD classification can be considered a universally standardised method of classification and recording allowing consistency across studies (Elfrink et al. 2015). The examiner (E.J.) was able to conduct the MIH examination in a standardised, calibrated and efficient manner in all children included in the study (Elfrink et al. 2015; Ghanim et al. 2015).

There are limitations to this study, including a total of 73 participants, below the recommended number of participants for the prevalence of MIH (Elfrink et al. 2015). However, this is the first cohort of children with T1D examined for MIH prevalence. There was no control group in this study or any comparative data for the prevalence of MIH in healthy children. Despite the limitation, this study provides additional insight into potential aetiological factors of MIH by describing the prevalence of MIH in a cohort of children with T1D. Unfortunately, only 19.18% of participants were diagnosed with T1D under 4 years of age, which is when potential disruption to amelogenesis would be anticipated to be a contributory factor to the development of MIH. Hypomineralisation in other primary and permanent teeth was recorded during examination. However, many participants were in the mixed dentition and a decision was made to only report first permanent molars and permanent incisors, required to make a formal diagnosis of MIH. Longitudinal studies evaluating hypomineralisation on primary and permanent dentition for children diagnosed with T1D before four years of age may further our understanding of any potential causative associations to the development of MIH.

## Conclusion

Within the limitations of the present study in a convenience sample of children with T1D, it has been shown that children with T1D revealed a higher prevalence of MIH at 19.18%, when compared to estimated prevalence rates of 13.5% in healthy children globally. There were no significant associations noted between MIH and risk factors including average glucose levels over the previous 3 months (*p* > 0.1). Future large-scale longitudinal studies are necessary, particularly with larger samples of children who were diagnosed with TID at a young age to explore whether there is any association explaining the greater prevalence of MIH in children with T1D.

## Data Availability

This manuscript has associated data in a data repository. All data included in this manuscript are available upon request by contacting with the corresponding author.
